# Acute pancreatitis experimental models, advantages and disadvantages

**DOI:** 10.1007/s13105-025-01091-w

**Published:** 2025-05-17

**Authors:** Genaro J. Rosales-Muñoz, Verónica Souza-Arroyo, Leticia Bucio-Ortiz, Roxana U. Miranda-Labra, Luis E. Gomez-Quiroz, María Concepción Gutiérrez-Ruiz

**Affiliations:** 1 Posgrado en Biología Experimental, Universidad Autónoma Metropolitana-Iztapalapa, Mexico City, Mexico; 2 Departamento de Ciencias de La Salud, Área de Medicina Experimental y Traslacional, Universidad Autónoma Metropolitana-Iztapalapa, Mexico City, Mexico; 3 Laboratorio de Medicina Experimental, Unidad de Medicina Traslacional IIB/UNAM, Instituto Nacional de Cardiología Ignacio Chávez, Mexico City, Mexico

**Keywords:** Pancreatitis, Animal models, Laboratory animals, Basic amino acids

## Abstract

Acute pancreatitis represents a severe health problem, not only because of the number of people affected but also because of the severity of its clinical presentation that can eventually lead to the death of patients. The study of the disease is complex, and we lack optimized models that can approach the clinical presentation in patients, in addition to the significant vulnerability of the organ itself. In the present work, we undertook the task of reviewing and analyzing the experimental methods most currently used for the induction of acute pancreatitis, emphasizing the advantages and disadvantages of each model and their delimitation based on experimental objectives. We aimed to provide an actual and quick-access guide for researchers interested in experimental acute pancreatitis.

## Introduction

**Fig. 1:**
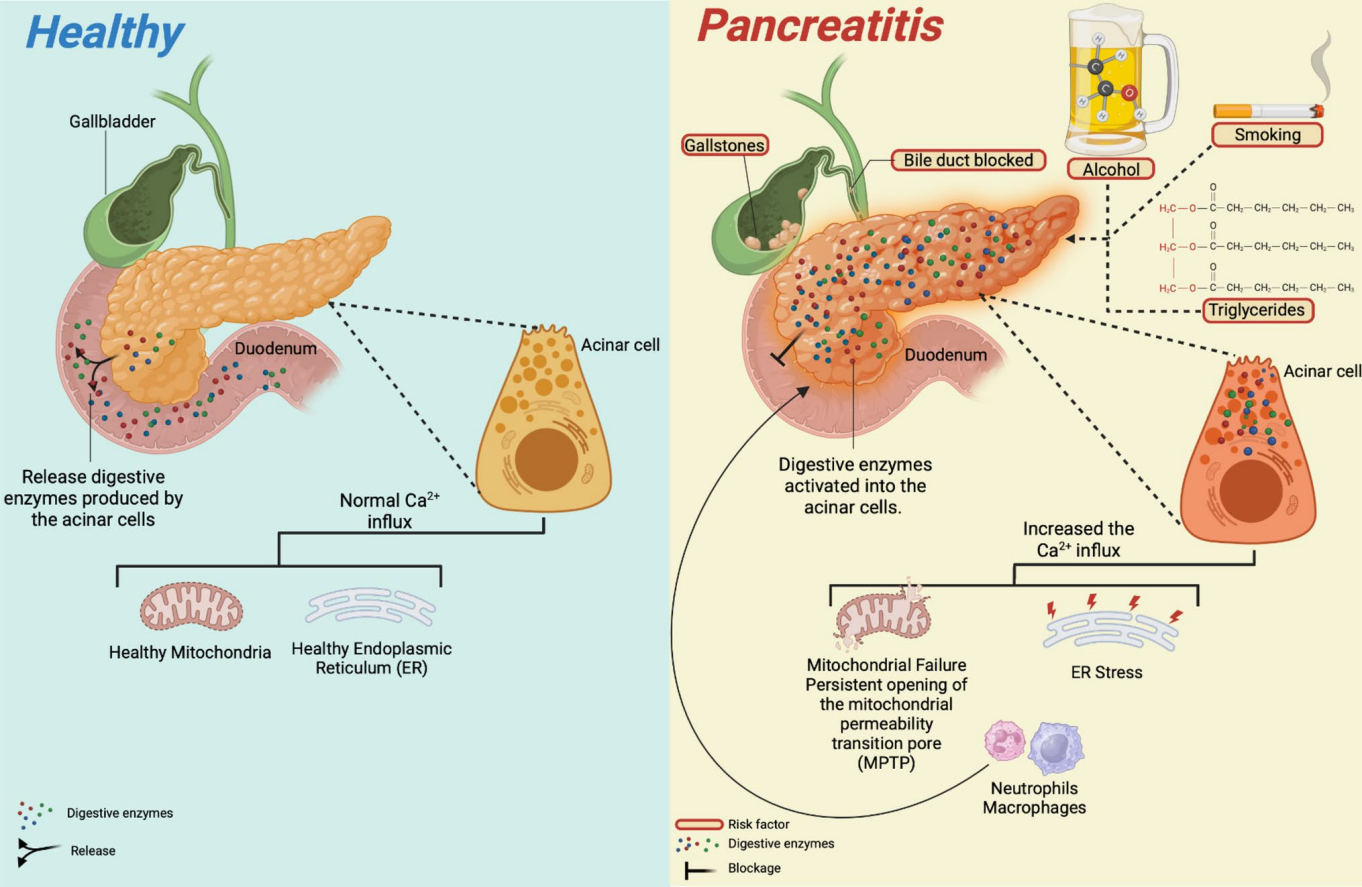
Pathophysiology of acute pancreatitis. The most frequent etiological agents that trigger the development of Acute Pancreatitis (AP) are biliary cholelithiasis, alcoholism, smoking, hypertriglyceridemia, and obstruction of the pancreatic duct. Regardless of the mechanism, the pathophysiology of AP leads to a deficiency in pancreatic excretion processes, preventing the exocytosis of zymogen granules (GZ) that contain digestive enzymes produced by acinar cells. Sustained cellular Ca2+ signaling leads to the development of aberrant exocytosis of GZ, which can co-localize with lysosomes in the plasma membrane, leading to intracellular activation of digestive enzymes that cause damage to different organelles such as the endoplasmic reticulum (ER), and mitochondria, causing reticulum stress (ER stress) and a persistent opening of the mitochondrial membrane permeability pore (MPTP), respectively. The secretion of chemoattractant factors causes the recruitment of immune system cells (neutrophils and macrophages) in the pancreas, which can promote tissue inflammation and culminate in a severe systemic inflammatory response. Created with Biorender.com

Acute pancreatitis (AP) ranges from 4.9 to 80 cases per 100,000 persons annually [1]. There are multiple causes and pathological conditions potentially associated with AP. It is an inflammatory disease within the pancreas, caused mainly by cholelithiasis, hypertriglyceridemia, or excessive alcohol consumption. The pathogenic mechanism of AP is by inappropriate activation of trypsinogen and destruction of secretory cells followed by systemic release of cytokines and inflammatory mediators, causing the activation of inflammatory cells, fever, and multiple organic failures [2–4] (Fig. 1). In most patients, about 85% to 90%, the disease takes a mild course, self-limited, where moderate fluid resuscitation, management of pain and nausea, and early oral feeding result in rapid clinical improvement [5, 6].

Meanwhile, necrotizing pancreatitis, which accounts for the remaining 10% to 15% of cases, follows a grave clinical course that is more often accompanied by local/systemic complications associated with multiple organ dysfunction syndrome, a life-threatening disease with hospital mortality rates of about 15% [7, 8]. Diagnosis of AP is based on clinical presentation (acute pain attack in the upper abdomen spreading to the back), laboratory test (serum lipase and or amylase levels are three or more times higher than normal values), and imaging findings characteristics for AP (computer tomography, magnetic resonance imaging, ultrasonography). It requires two of the three criteria to consider: AP [3]. According to the Atlanta classification, based on organ failure as well as local and systemic complications, the severity of AP is divided into three levels: mild, moderately severe, and severe [9]. Despite the considerable progress made over the past decades, the therapy of AP remains supportive, as there are no specific treatments that can alter the course of the disease. This lack of target therapy is mainly due to our incomplete understanding of molecular processes of the underlying mechanism of AP and reliable biomarkers to diagnose severe acute pancreatitis early. Animal model systems of AP could help determine the disease's mechanism and find potential biomarkers that predict the disease onset and grade.

Cellular events driving the pathogenesis of acute pancreatitis include aberrant calcium signaling [10–12], mitochondrial dysfunction [13, 14], premature trypsinogen activation within the acinar cells and macrophages [15–17], endoplasmic reticulum (ER) stress, impaired unfolded protein response (UPR), and impaired autophagy [13, 18, 19]. Common acinar cell toxins like alcohol, nicotine, and bile acids trigger these events [20]. Common acinar cell insults like ethanol, nicotine, and bile acids trigger these events. Intraductal actions, such as increased pressure caused by ductal obstruction, luminal acidification, and ductal cell exposure to bile acid, can also indirectly trigger these dealings. The crosstalk between acinar cells and the immune cells prolongs the inflammatory response, aggravating the disease [20].

The pathophysiologic event of experimental acute pancreatitis consists of the activation of pancreatic enzymes within acinar cells; the release of these activated enzymes in the interstitium leads to autodigestion of the pancreas that triggers an inflammatory response mediated by the release of cytokines and chemokines from the damaged acinar cells, which recruit immune cells to the site of the injury. These immune cells release additional inflammatory mediators and reactive oxygen species, exacerbating tissue damage and inflammation. In some cases, inflammation is localized in the pancreas, but releasing inflammatory mediators could lead to a systemic inflammatory response that results in multiple organ dysfunction [21]. An overview of AP models will be discussed, focusing on advantages and limitations.

## Animal models

Experimental animals have long been used in biomedical research and have contributed to finding solutions to biological and medical issues. Laboratory animal models have been developed to study human diseases; as a result, they have improved human health by helping scientists better understand the condition's physiopathology and molecular mechanisms, thus more accurately identifying molecular targets of drug treatment. Various animal models are still being developed and utilized for laboratory research purposes. Due to the anatomical localization of the pancreas and the practical challenges of obtaining human pancreatic tissue during different stages of the inflammatory process in humans, our understanding of the pathogenesis of all early events at the molecular and cellular level mainly relies on data from experimental animal models.

**Fig. 2:**
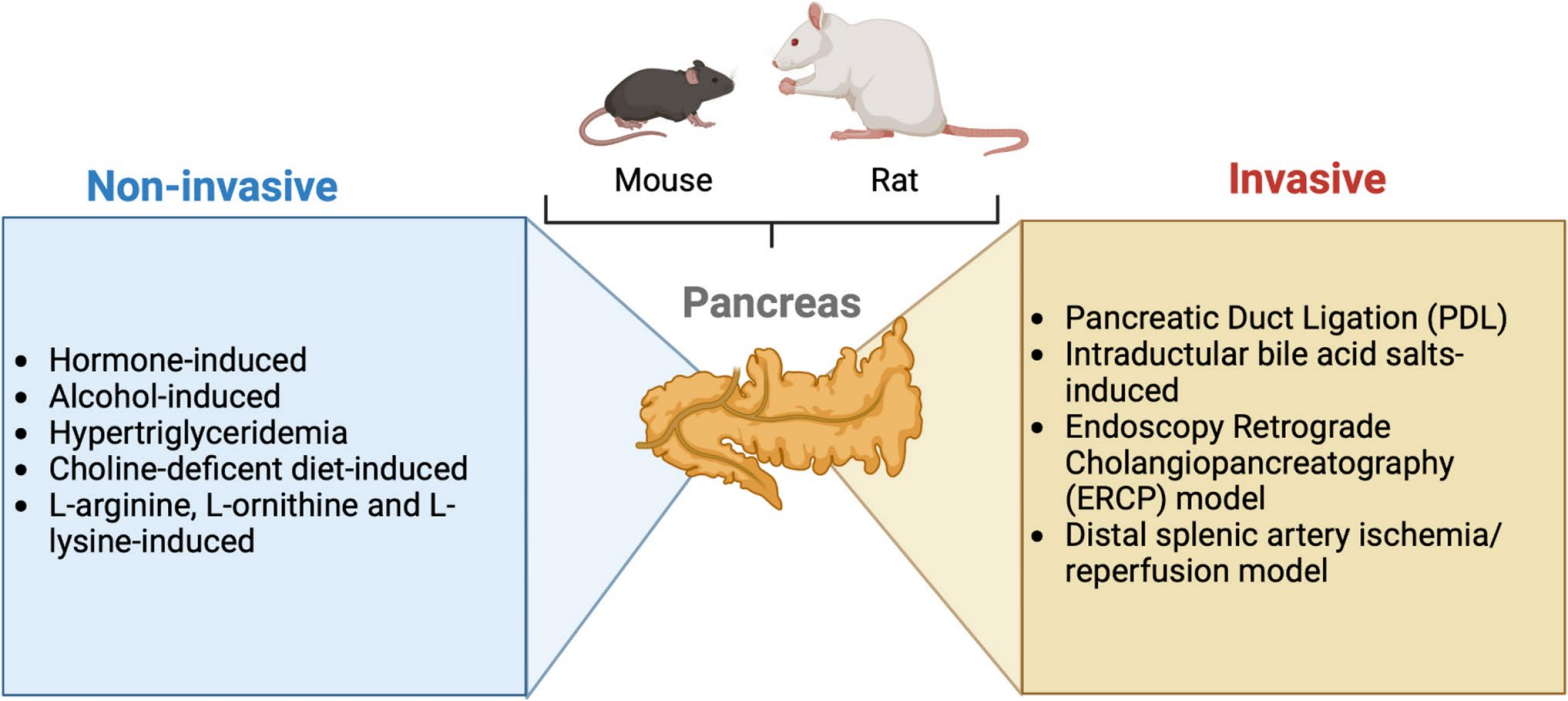
Acute pancreatitis animal models. Animal models of acute pancreatitis (AP) are an essential research tool in translational medicine. The development of different experimental models allows the generating conditions analogous to the various etiologies that generate human AP. These can be divided into non-invasive models, which include those induced by hormones, alcohol, hypertriglyceridemia, choline-deficient diet, and basic amino acids ( L-arginine, L-Ornithine and L-lysine) as well as invasive models such as pancreatic duct ligation, intraductal bile salt injection, the endoscopic retrograde cholangiopancreatography (ERCP) model and the distal splenic artery ischemia/reperfusion model. Created with Biorender.com

Selecting an animal model for AP research is challenging, as all models have some limitations. A lot of factors should be taken into consideration when choosing an ideal animal model for AP trials. One of the most important criteria is the proper selection of models for resemblance between animal species and humans regarding physiological and pathophysiological aspects [22]. Models of AP study the disease at the cellular and whole-animal level and in a range of species. Also, the proper model selection is determined by the pathophysiological mechanism of interest, the disease phase of interest, or the effectiveness of new therapeutic procedures [23]. Many species, including dogs, cats, guinea pigs, and even zebrafish, have been used due to the low cost, high reproducibility of rodent models, and the available strains with genetic deletions; mice and rats are used most [24, 25]. Although rodent models are used most often, their differences from the human exocrine pancreas concerning various factors, such as digestive enzyme content, should be recognized [24, 26]. Theoretically, a reliable experimental model must be efficiently produced to reproduce the disease in terms of etiology, histology, symptomatology, and effectiveness in treatment, which means the AP models should mimic remarkable pathophysiological changes in human pancreatitis [27, 28]. In this review, we will divide the models of AP into non-invasive and invasive (Fig. 2).

## Non-invasive models of acute pancreatitis

The main advantages of the non-invasive models include their lack of requirements for surgery or complicated manipulations.

**Fig. 3:**
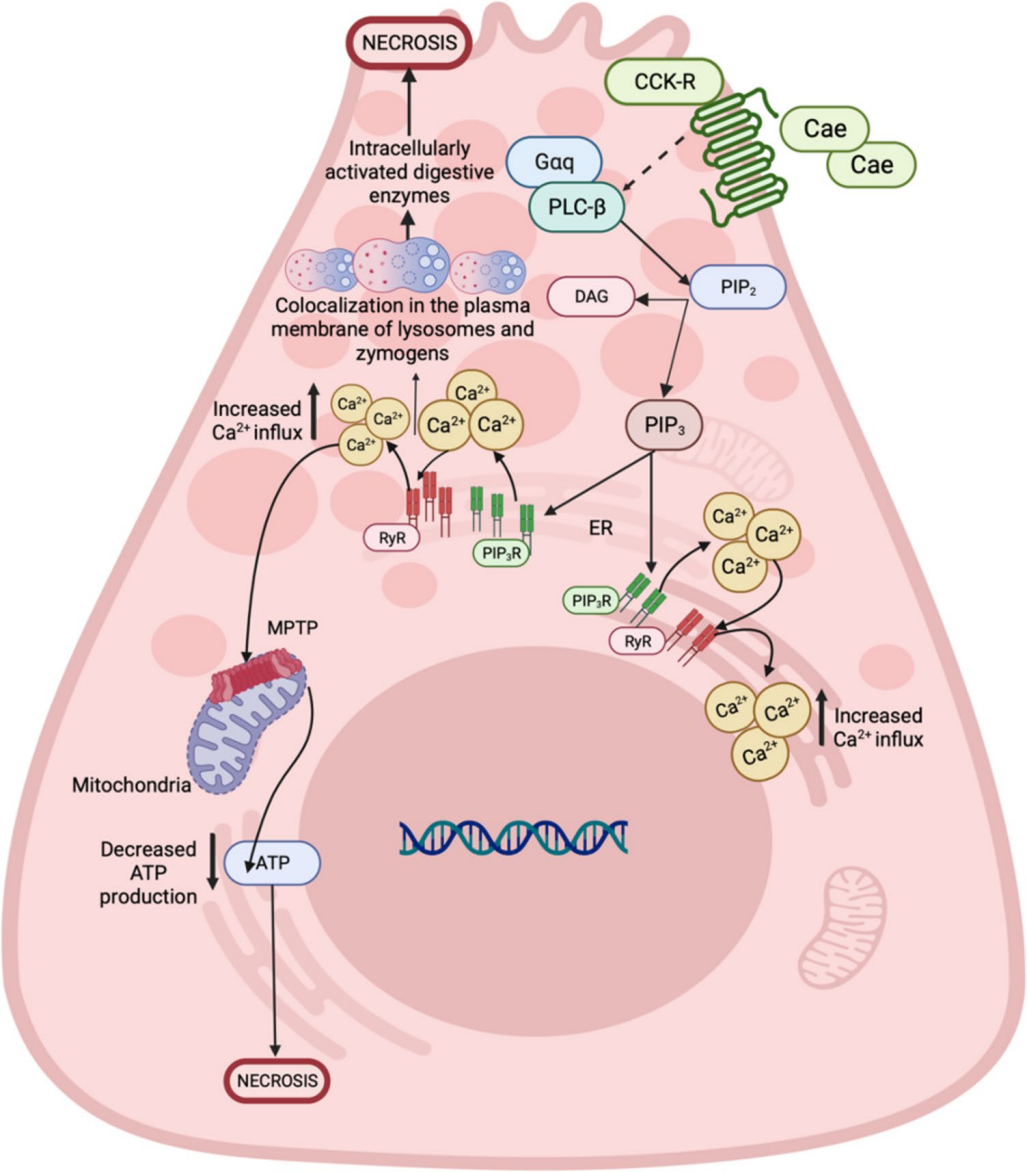
Cellular pathogenesis of acute pancreatitis induced by cerulein. The cerulein (Cae)-induced acute pancreatitis (AP) model is currently the most used model that resembles human AP. Cerulein (Cae) is a peptide analog of cholecystokinin (CCK); for instance, it can bind to cholecystokinin receptors (CCK-R) present in the membrane of pancreatic acinar cells; these receptors are classic seven transmembrane receptors coupled to trimeric G proteins (G proteins). Activation of these receptors leads to stimulation of Gαq and an increase in the activity of phospholipase C beta (PLCb), which converts the membrane phospholipid: phosphatidylinositol,4,5-bisphosphate (PIP_2_) into diacylglycerol (DAG) and phospho-inositol. 1,4,5-triphosphate (PIP_3_). PIP_3_ interacts with PIP_3_ receptors (PIP_3_R), present in the endoplasmic reticulum (ER), releasing Ca2+ into the cytoplasm, which can induce the opening of the mitochondrial permeability transition pore (MPTP) and cause a decrease in ATP levels. In addition, an increase in the exocytosis of zymogen granules is also observed, causing their accumulation in the apical area of ​​the acinar cell. This initiates the premature activation of digestive enzymes due to membrane colocalization between lysosomes and zymogen granules, ending in necrosis of the acinar cell. Created with Biorender.com

1. **Caerulein-induced AP model or hormone-induced**: Caerulein (Cae)-induced AP model is the most widely used AP model, as it is highly reproducible and cost-effective [28]. Normal pancreatic metabolism is associated with physiological concentrations of secretagogues [29, 30]. AP results from an excess availability of secretagogues, which leads to a high secretion of pancreatic digestive enzymes [2, 8]. The Cae model is among several animal models of experimental acute pancreatitis exhibiting biochemical, morphological, and pathophysiological similarities to various aspects of human pancreatitis [31, 32]. Cae-induced pancreatitis is like human edematous pancreatitis, manifesting with dysregulation of digestive enzyme production and cytoplasmatic vacuolization. The death of acinar cells, edema, and infiltration of inflammatory cells into the pancreas is one of the best-characterized and widely used experimental animal models [33]. Cae is a hormone analogous to cholecystokinin (CCK) that binds to the acinar cell-restricted CCK-A receptor, thereby inducing pancreatitis through intrahepatic activation of digestive enzyme production and cytoplasmic vacuolization [28]. It was first isolated from skin extracts of the Australian green tree frog [34]. Cae acting through the CCK receptors yields exaggerated stimulation of acinar cells, leading to trypsinogen's prematuration [32] (Fig. 3).

2. Cae-induced pancreatitis is a widely used experimental model to study acinar cell physiology and the early stages of pancreatitis. When given 10–100 times greater than a physiologic equivalent, it has been used to cause acute pancreatitis successfully [31]. Intravenous, subcutaneous, or intraperitoneal injection routes may induce acute pancreatitis; this method may increase proteolytic enzyme secretion to levels that cause pancreatic acinar autolysis. One hour after the infusion of Cae, progressive interstitial edema develops and reaches a maximum after 12 h [32, 35]. The dose of Cae may be gradually increased. Pancreatic enzymes in the blood circulation, pancreatic inflammation, acinar cell necrosis, and fat necrosis can be observed following a two-fold increase in the Cae dose. This model is generally self-limiting without multiple organ dysfunction syndrome and lethality, which could be considered its most significant limitation [36], as it does not mimic severe forms of human AP. To address this limitation, Cae is combined with other compounds to achieve increased severity of Cae acute pancreatitis, i.e., lipopolysaccharide (LPS) [37]. Infusion of enterokinase (EK) after Cae administration causes pancreatitis necrosis, hemorrhage, and high mortality rates in mice and rats [38, 39]. Combining methods to generate more prominent damage has also been widely used, such as combining Cae with partial duct ligation, which allows the study of acute reversible changes and progressive fibrosis in the organ [16, 22, 40]. This highly reproducible model has been used to successfully cause acute pancreatitis in mice, rats, Syrian hamsters, and dogs [41, 42]. There may be differences among animal models in the development of interstitial edema, intracellular vacuolation, and the degree of necrosis. Differences have also been found in the role of autophagy and apoptosis in developing pancreatitis in the different animal models. Also, it has been used extensively in research settings, and the pathogenesis of acute pancreatitis induced by this agent is reasonably well understood. Mechanistically, this model is like pancreatitis caused by scorpion venom of the *Tityus* species or cholinergic toxins [43–45]. The advantages of this model are its non-invasiveness, low cost, rapid induction, high reproducibility, and broad applicability. Also, a great advantage is that it can be used in in vitro models [37].

3. **Alcohol-induced**: In the pancreas, the oxidative biotransformation of ethanol that generates acetaldehyde may be less critical than the non-oxidative, which produces fatty acid ethyl esters (FAEE) [46, 47]. Acetaldehyde has no acute effects on pancreatic acinar cells, even at 5 mM concentration, although it could activate pancreatic stellate cells and contribute to fibrosis [48]. The importance of non-oxidative metabolism in the pancreas has been known since Laposate and Lange (1985) found in an autopsy of an alcoholic patient that the concentration of FAEE in the pancreas was directly related to the concentration of alcohol in the blood [49]. Studies in isolated mouse pancreatic cells suggest that fatty acids and FAEE are linked to mitochondrial damage. There is a subsequent decrease in ATP and increased cytosolic calcium levels through failure to actively pump calcium out of the cytosol [50, 51].

Although one of the major etiologic factors of AP is alcohol, the mechanisms involved in alcoholic pancreatitis are incompletely understood [52]. However, it is well documented that AP induced by alcohol alone has been challenging to achieve and requires prior sensitization with other factors, such as exocrine hyperstimulation, to allow significant pancreatic damage [53–55]. The most used are secretagogues, LPS, or palmitoleic acid [56, 57]. The alcohol-induced model could be performed via intravenous, oral, intraperitoneal, and intraductal administration [54, 58]. The morphology of the AP varies according to the animal model, the dose of ethanol administration, and the pre-sensitization agents used [59]. Pandol et al. (1999) demonstrate that ethanol feeding sensitized rats to pancreatitis and that this sensitization involved NF-κB activation [60]. Yuan et al. (2022) reported an amplified protein kinase D (PKD) signaling that plays an essential role in mediating the effect of alcohol abuse on the pathological features of pancreatitis [61]. Also, alcohol increases pancreatic duct permeability, decreases pancreatic blood flow and microcirculation, diminishes pancreatic oxygen consumption, and induces oxidative stress [62, 63]. Among the most widely used models to study acute alcohol pancreatitis are mice and rats fed with Lieber-DeCarli ethanol diet for 6–8 weeks, then animals received up to 4 intraperitoneal (IP) injections of Cae at one-hour intervals and euthanized at different periods after the first IP injection, depending on the objective of the study [64–66]. Ethanol and other alcohols sensitized the acinar cells to Cae-induced trypsin and chymotrypsinogen activation [67, 68]. However, when only ethanol is used, neither acute pancreatitis nor zymogen activation is induced in experimental models of acute pancreatitis, a disadvantage of this model [68]. It mimics ethanol-induced liver damage but not alcohol-induced pancreatitis, and some authors consider a lack of correlation with clinical situations [69]. Werner et al. (1997) studied the acute effects of ethanol on the pancreas by the infusion of non-oxidative products of ethanol metabolism, such as FAEE, in rats. They observed increases in pancreatic edema formation, pancreatic trypsinogen activation, and vacuolization of acinar cells. Authors concluded that FAEE at concentrations found in human plasma produces an acute pancreatitis-like injury in rats, providing direct evidence that FAEE can have organ-specific toxicity and could play an essential role in acute alcohol-induced damage to the pancreas [70]. Ethanol and other alcohols have been shown to sensitize acinar cells to Cae-induced various forms of pancreatitis and trypsin activation *in vivo* and to intracellular trypsinogen activation *in vitro*. Thus, one of the major mechanisms of acute pancreatitis in alcoholics involves connections between alcohol, intracellular hypercalcemia, and trypsinogen activation [71]. Several animal models, including rats, cats, and dogs, have been used.

The cellular and molecular mechanisms of ethanol-mediated AP remain partially identified. It is reported [72] that ethanol (50 mM) increases ROS, which is related to oscillations in cellular Ca^2+^ release from intracellular stores [73] in freshly isolated mouse pancreatic acinar cells stimulated with CCK-8. The inhibition of the alcohol dehydrogenase with 4-methylpyrazole did not affect the peak of Ca^2+^ release into the cytosol, but the"steady-state level"was significantly reduced, as was the rate of decay of Ca^2+^ to basal values, indicating that the noxious effects of ethanol in the acinar cell are partially dependent on ethanol biotransformation. However, the alcohol dehydrogenase enzyme does not produce ROS; the source of these toxic species awaits elucidation, but it is clear that ROS and Ca^2+^ are undoubtedly involved in ethanol-induced AP, reinforced by the amylase secretion by acinar cells [74].

**Fig. 4:**
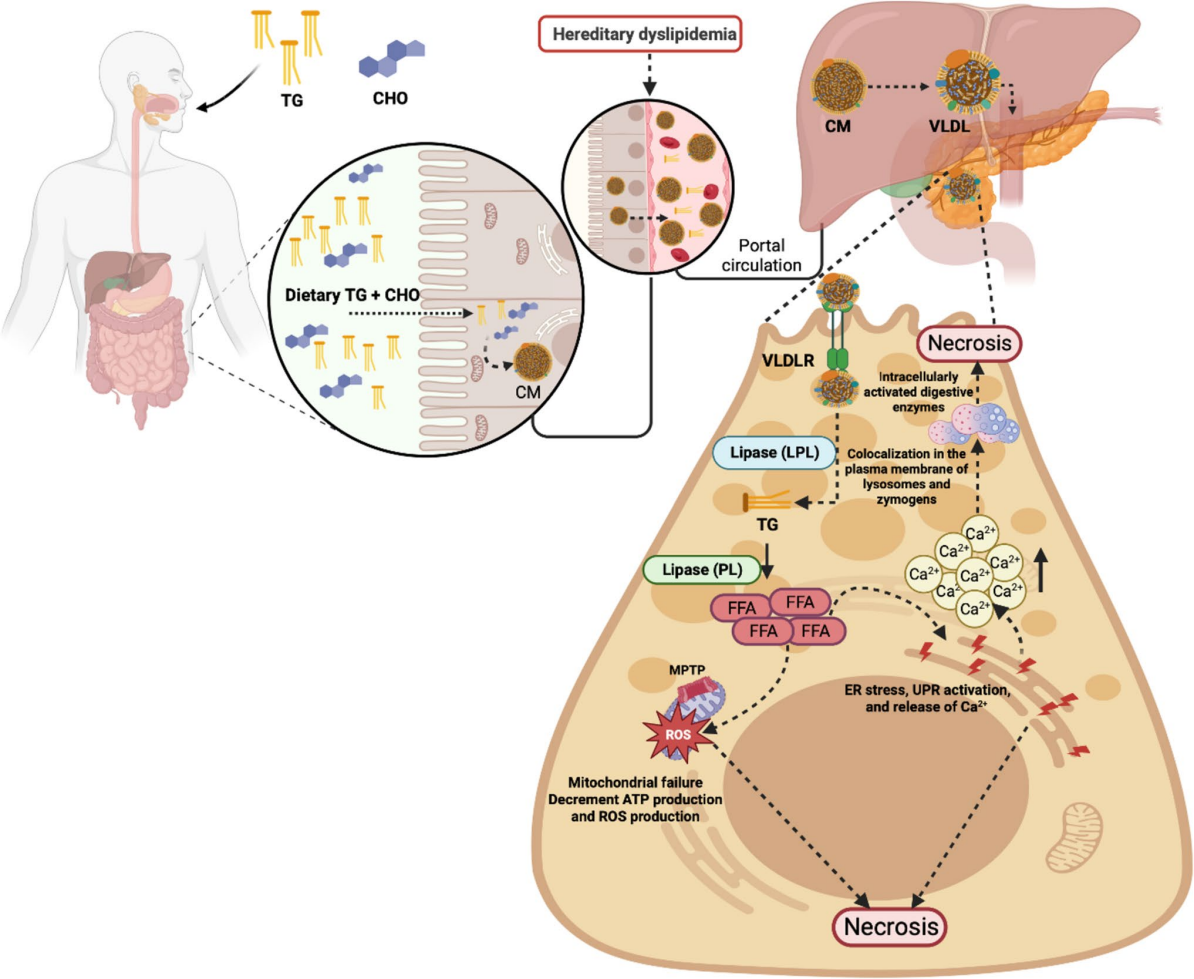
Cellular pathogenesis of acute pancreatitis induced by hypertriglyceridemia. Hypertriglyceridemia (HTG) is considered the third cause of the development of acute pancreatitis. However, the mechanism by which HTG leads to the development of pancreatitis needs to be clarified; evidence suggests the following mechanism. The intestine absorbs dietary triglycerides (TG) and cholesterol (CHO), and they are transformed into chylomicrons (CM) that subsequently travel to the liver, where the hepatocytes package them into very low-density lipoproteins (VLDL), which will be captured by VLDL receptors (VLDLR) in the acinar cells. The lipoprotein lipases (LPL) in the acinar cell convert VLDL into TG and CHO; TG´s are the substrate of pancreatic lipase (PL) and lead to free fatty acids (FFA) as a product. When the regulation of FFA metabolism is inadequate, it can cause massive accumulation of FFA in the pancreas, which is cytotoxic *per se*, and trigger an inflammatory reaction, release intracellular Ca^2+,^ and decrease ATP levels by opening the mitochondrial permeability transition pore (MPTP), and by the inhibition of mitochondrial complexes I and V, It can also affect the endoplasmic reticulum (ER), which in response to stress releases Ca^2+^, causing the aberrant release of zymogen granules, which can colocalize with lysosomes and the intracellular activation of digestive enzymes, which lead the acinar cell to necrosis. Created with Biorender.com

4. **Hypertriglyceridemia-induced**: Lindkvist et al. have reported that the risk of AP was about 5% when serum TG level > 1000 mg/dl and increased dramatically up to 10–20% when the serum TG level > 2000 mg/dl [75]. Hypertriglyceridemia-induced acute pancreatitis (HTG-AP) has become the second major cause of AP [76]. Although the association between HTG and AP is well established, HTG as a risk factor for AP in the general population needs to be better identified; the current international consensus strongly suggests that AP patients with serum triglyceride levels > 1000 mg/dl have HTG-AP [76]. The existing literature on HTG-AP mainly focuses on the analysis of clinical characteristics, pathophysiology, and epidemiology, and there is a need for more mechanistic research, which could be due to the need for an appropriate model of HTG-AP [77] (Fig. 4). The Lipoprotein Lipase (LPL) activity of mice and rats is so high that simply feeding a high-fat diet cannot establish an ideal HTG animal model (TG level > 1000 mg/dl) [78]. Some studies in genetically modified mice include LPL-deficient mice [79, 80], LPL-deficient cats [81], and other species that exhibit high TG levels.

Models for establishing inducible HTG-AP first must be induced through the diet and other methods; then, AP is induced by injection of drugs, commonly Cae. Chronic HTG includes Triton WR 1339 injection intravenously or intraperitoneally [82]. Triton WR 1339, a non-ionic detergent, can increase the concentration of TG plasma by inhibiting LPL activity [83]. Harnafi et al. (2007) injected Triton WR1339 into the abdominal cavity of rats [84]. As a result, the serum TG levels of rats in the model group were more than 20 times higher than those in the control group; the disadvantage of this method is that the drug cost is relatively high, and the TG levels will fluctuate with time after the model has established [85]. Pan et al. (2017) reported that Poloxamer 407 (P-407), a hydrophilic triblock copolymer comprised of polyoxyethylene and polypropylene units, has been reported to induce HTG with little side effects [78]. P-407 increases serum TG concentration by directly inhibiting the activity of LPL and hepatic lipase, combined with the capillary wall [86]. Saja et al. (2015) reported that P-407 could raise serum TG to 4000 mg/dl after being treated with P-407 for 28 days. P407 could elevate ApoCIII-serum levels, which affected TG metabolism and induced hypertriglyceridemia [87]. Pan et al. (2017) reported a novel animal model of HTG-AP that mimics the physiological, histological, and clinical features of human HTG-Ap and could help promote therapeutic strategies and advance the mechanism research on HTG-AP [78]. C57BL/6 male mice were treated with P-407 at 0.5 g/kg body weight [86, 88]. One intraperitoneal injection of P-407 established the transient HTG model, the short-term HTG model was based on seven consecutive dosing days, and the long-term HTG model was set up via 28 consecutive dosing days [88]. AP was induced by ten IP injections of Cae B.W. in PBS at hourly intervals. In the study, the authors reported that serum triglyceride levels caused by P-407 were elevated in a dose-dependent manner. The pancreatic and pulmonary injuries were much more severe in HTG mice than in normal mice when injected with a conventional dose of Cae (50 μg/kg); the severity of AP was positively correlated with the duration and extent of HTG. In addition, they reported that low-dose Cae (5 μg/kg) could induce pancreatic injury in HTG mice. At the same time, normal mice had no apparent pathological injury [78]. Also, they reported that HTG led to the increased infiltration of macrophages and neutrophils in the pancreatic tissues of mice. In this way, P-407 joint Cae can build a stable and controllable mouse model of severe HTG-AP [78]. Although the hyperlipemia in models established by Triton WR 1339 injection and P-407 injection is not consistent with that in the naturally occurring hyperlipidemia state, the time to develop the model is short, the operation is simple and controllable, and the TG levels of animals are stable and higher than those of the animal models established by the feeding method; therefore, this method is also widely used at present [85].

5. **Diet-induced**: Choline-deficient diet enriched with ethionine- In the 1930 s, a diet deficient in the essential nutrient choline, a member of the vitamin B complex and an important component of cell membranes, damaged the pancreas [28, 89–91]. Acute pancreatitis in rats treated with ethionine was first reported in 1950 by Farber and Popper and Goldberg et al. Since then, ethionine-induced pancreatitis has been shown to occur readily in other species of experimental animals, including the mouse, hamster, cat, dog, and monkey [92–96]. The lesions produced mainly focal necrosis and atrophy with regenerative attempts of the acinar cells. So, the ethionine model has been used frequently to investigate the pathogenesis of acute pancreatitis and to study the regenerative power of the pancreatic parenchymal cells. Lombardi et al. (1975) reported acute hemorrhagic pancreatitis with observed death in 100% of mice of acute necrotizing pancreatitis with diffuse necrosis of the fat tissues in mice after four days of feeding with a choline-deficient diet enriched with ethionine (CDE) [92]. Acute severe necrotizing pancreatitis with fat necrosis is induced in mice by DL-ethionine fed a choline-deficient diet [92]. This model was initially developed using young female mice; males can be used after estrogen administration, and the diet has similar effects in rats. The amount of injury produced by the CDE diet depends critically on the sex, age, and weight of the mice. Although pancreatitis induced by the CDE is more severe than that caused by secretagogues, the models share many standard features, including blocked secretion of digestive enzymes and the formation of cytoplasmic vesicles containing lysosomal hydrolases colocalized with digestive enzymes [90]. Induction of pancreatitis by the CDE diet has also been associated with the activation of intrapancreatic proteases and characteristic vascular changes. Although it is a non-invasive model, the diet is costly and requires on-site protocol standardization and careful monitoring of dietary intake. Therefore, variation among animals could be high, making the use of many animals in each group necessary for meaningful results. This diet model is particularly suitable for studying the potential for new therapeutic substances, as CDE diet-induced pancreatitis shares many characteristics with severe human acute pancreatitis [97].

6. Basic amino acid-induced

- L- Arginine- L-arginine-induced AP is currently the most used amino acid-induced AP model in rats and mice. The effect of L-arginine on the pancreas has been extensively investigated since 1984, when a single IP injection of L-arginine at 5 g/kg led to a long-lasting pancreatic adenocarcinoma (PAC) and adipose tissue necrosis in the rat pancreas, without affecting islets and other organs [98]. Higher doses (7.5 g/kg) of L-arginine can be lethal for experimental animals, whereas a 2.5 g/kg dose only causes mild pancreatic injury. The mechanism of arginine-induced AP remains to be fully understood [99, 100]; however, its toxicity in the pancreas is probably due to inhibition of protein synthesis, excessive nitric oxide production, or lipid peroxidation [101]. Excessive arginine and other basic amino acids could suppress ornithine decarboxylase, the rate-determining enzyme in polyamine synthesis [32]. The resultant reduction in polyamine levels retards nucleic acid synthesis, which may interfere with protein synthesis. Pancreatic tissue is particularly vulnerable to this toxicity because of its active protein metabolism [102]. In a rat model of L-arginine-induced pancreatitis, partial inhibition of L-arginase activity, an enzyme that converts L-arginine to L-ornithine and urea, ameliorates pancreatitis, indicating a role of one of its metabolites in the induction of pancreatitis [103]. Further, at high doses, L-ornithine has also been reported to cause acute pancreatitis [104, 105]. L-arginine is a substrate for nitric oxide synthase and is known to induce both nitrosative and oxidative stress. L-arginine pancreatitis is ameliorated by treatments that reduce oxidative stress [106–108]. Arginine-induced pancreatitis is a relatively non-invasive AP animal model. A single arginine injection is usually strong enough to induce acute necrotizing pancreatitis, although sometimes multiple injections may be required [109]. The degree of severity of this animal model depends on the dose of arginine and the time of exposure. Usually, interstitial edema, neutrophil infiltration, acinar cell degranulation, and elevated amylase are observed when arginine is administered in low doses [98–100, 103, 105, 107, 108]. One of the disadvantages of arginine-induced pancreatitis is its lack of clinical relevance.
- L-Ornithine- Rakonczay et al. (2008) developed a simple, noninvasive, reproducible model of acute necrotizing pancreatitis by intraperitoneal injection of 3 g/kg L-ornithine showing typical laboratory and morphologic signs observed in the human disease. L-ornithine-induced pancreatitis is superior to the L-arginine-induced model in that it produces a much more severe illness with massive edema without the confounding effects of possible excessive NO synthesis. IP administration of 4–6 g/kg L-ornithine killed the rats within hours before pancreatitis could develop [110]. These animals'deaths may result from effects on the central nervous system. Authors suggest that L-arginine-induced pancreatitis is due to the metabolism of L-arginine to L-ornithine by arginase and is not caused by its metabolism to L- citrulline by NOS. That is, L-arginine administration results in a more significant increase in blood concentration of ornithine than citrulline, and administration of L-ornithine but not L-citrulline causes severe pancreatitis [110]. Biczo et al. (2010) found that pancreatic polyamine catabolism was activated in L-ornithine-induced AP and tried to ameliorate it with metabolically stable polyamine analogs, which turned out to be ineffective [111]. Several molecular features that have been observed in other models of pancreatitis were observed with the administration of L-ornithine, among them intrahepatic activation of the digestive enzyme trypsinogen, degradation of IκB proteins associated with activation of NF-κB, increased production of interleukin-1β, and evidence of oxidative stress [99]. Ornithine-induced AP is not possible in mice as L-ornithine (2 × 4 g/kg) produced death in treated mice within a few hours [105].
- L-Lysine- Biczó and collaborators. (2011) demonstrated that large doses of IP-injected L-lysine in Wistar rats induce acute necrotizing pancreatitis. Induction of severe AP was most effective with IP injection of 2 g/kg L-lysine; L-lysine administration causes early pancreatic mitochondrial damage that impairs the ATP synthase activity of pancreatic mitochondria [112]. This occurs early in the development of the disease and is followed by the activation of trypsinogen and the proinflammatory transcription factor NF-κB and acinar cell death through apoptosis and necrosis, which are commonly thought to play an important role in the development of AP [45, 55]. The authors pointed out that L-lysine selectively damages pancreatic mitochondria but does not affect liver mitochondria. Méndez et al. (2021) reported that administering L-lysine and alloxan induces acute necrotizing pancreatitis in rats and consider this model economical and reproducible [27].
- L-Histidine- Zhang et al. (2018) reported that mice treated IP with histidine 2 × 4 g/kg (1 h apart) can induce progressive pancreatic necrosis and associated lung injury. These authors also reported that L-arginine, L-ornithine, and histidine produced AP and reported significant differences in the mechanism of pancreatic injury [105]. Metabolism of L-arginine to L-ornithine is involved in the pathogenesis of L-arginine-induced AP. It is well known that hereditary diseases of branched-chain amino acid metabolism will significantly increase the risk of AP in humans [113]. Therefore, the primary amino acid-induced AP model could also have links to human disease. AP caused by amino acid overdose is rare in humans [114]. A potential limitation of L-histidine-induced AP is the low solubility of L-histidine in physiological saline, which requires more significant amounts of liquid for injections of higher concentrations of amino acid, and it has been a limitation to establish the model in rats [45].

## Invasive models of acute pancreatitis

The highly variable severity of acute pancreatitis associated with the non-invasive models and the ill-defined temporal relationship of onset have led to the development of invasive models to overcome these problems. Moreover, the etiology of the non-invasive models has minimal relevance to the clinical situation. Most invasive animal models of acute pancreatitis simulate mechanisms relevant to gallstone-induced AP, one of the most common etiologies of pancreatitis observed in clinical cases [69]. The question of how gallstones cause pancreatitis during their passage through the common bile duct was addressed experimentally more than 100 years ago. It prompted the creation of several animal models of pancreatitis [90]. Pancreatitis is frequently induced in animals by surgical ligation of the bile duct in combination with the pancreatic duct or by introducing bile acids into the pancreatic duct [115]. Although these preparations may not be relevant to the human disease onset mechanism, they have become important models for studies of the pancreatic inflammatory response and therapeutic agents designed to reduce disease severity and necrosis [90].

7. **Pancreatic Duct Ligation (PDL)**- Acute pancreatitis may be induced by ligating the distal bile duct at the level of the duodenum [69]. Surgical ligation of the pancreatic duct alone has not successfully induced AP. Furthermore, anatomical differences between species affect the development of the AP, so the changes observed in the pancreas after pancreatic duct ligation vary depending on the animal used [22]. PDL-induced AP in mice is associated with systemic inflammation, acute lung injury, multiorgan dysfunction, and death [116]. Although it could be used in AP, this model is generally used to produce chronic pancreatitis. The duct obstruction model mimics gallstone obstruction-induced AP in the clinical setting. The surgical manipulation is simple, requiring either ligation of the common biliopancreatic duct or pancreatic duct obstruction by vertical cannulation or inserting a balloon-tipped catheter [117]. When the pancreatic duct is ligated in rats, early changes in the duct can be observed at 1–5 h after ductal ligation, and 24 and 48 h findings such as pancreatic edema, inflammatory cell infiltration, hyperamylasemia, and so on are observed, compatible with acute pancreatitis. Several researchers use this model in rats to investigate the early stages of the disease pathogenesis. However, apoptosis is the primary mechanism of cell death in the rat PDL-AP model [118]. PDL in mice causes AP with systemic inflammation and multiorgan dysfunction syndrome, whereas biliary duct ligation and sham surgery do not [119]. This model mimics gallstone obstruction-induced AP with high mortality; thus, it could be used to investigate the pathogenesis of severe AP and test new therapies [45]. Due to its anatomical characteristics, the opossum has been used to produce severe acute hemorrhagic necrotizing pancreatitis [7, 8]. This laboratory animal has a pancreatic main duct, which drains into a long common bile duct, and both enter the duodenum at the papilla [120]. This model of duct opossum is helpful because it closely simulates the mechanical events that occur during or after gallstone passage. This cell model could identify acinar cell necrosis as the early point of injury in acute necrotizing pancreatitis [120] Sandoval et al. in 1996 show that the extent of pancreatic necrosis from duct-ligated opossums corresponds with the elevated levels of neutrophil infiltration. Neutrophils are considered to produce mediators such as hydrogen peroxide and oxygen that may be responsible for triggering necrosis [121]. Pancreatic outflow obstruction seems to be the critical pathophysiological event for the induction of AP [120], and it appears that bile reflux is not required for the pathogenesis of acute pancreatitis in this model [69]. The duct ligation-induced model mainly correlates with the clinical acute pancreatitis after Polya gastrectomy. The advantage of the duct ligation model is that it avoids artificial drug usage, which may produce unwanted systemic effects, as well as the theory relating to acute biliary pancreatitis with biliary pancreatic reflux [122]. However, the complexity that requires manual expertise, technical difficulty, high cost, limited reproducibility, and the analogy to chronic pancreatitis have made the duct ligation model infrequently used for investigating AP.
8. **Bile Acid Salts-induced AP**- The first experimental biliary AP model was established in 1856 by Bernard [123], who developed a method of retrograde injection of bile and olive oil into a canine pancreas through the ampulla of Vater. Since then, various bile salts have been reported to induce AP in different species. Biliary AP refers to the AP caused by biliary calculous diseases. It is the most common cause of AP and is associated with significant morbidity and mortality [124]. Obstruction of the common biliopancreatic duct by gallstones blocks the efflux of pancreatic zymogens, creates elevated pressure in the pancreas, and leads to bile reflux into the pancreatic duct [125–127]. Several experimental models have been designed to recreate this condition. Cannulation of the pancreatic duct has enabled researchers to apply the bile components into the pancreas of experimental animals in a more controlled way.

One of the most significant recent advances involves adapting this method to a mouse model, which opens the door to transgenic studies of biliary AP [123]. Ductal infusion of 100 μl of glycine-sodium-glycodeoxycholate (Na-GDC) in rats at concentrations 8.5 mM, 17 mM, and 34 mM causes progressive, severe, but non-lethal AP. The higher concentrations cause edematous and necrotizing pancreatitis, respectively [128, 129]. In 1992, a new model in rats using the combined actions of deficient concentrations of ductal infusion of Na-GDC and i.v. Cae injection (5ug/kg/h for 6 h) [129]. This model features a moderate onset of homogeneous mild pancreatic injury that lasts around 24 h and provides a potential for modulating severity and clinical relevance. The model is suitable for the evaluation of new therapeutic strategies. The Na-taurodeosycholate (Na-TDC) model was initially used in the 1980 s [130, 131] both with and without ductal infusion of various concentrations of trypsin. The model was employed frequently to explore the pathogenesis of AP with a commonly adopted regimen of 200 μl of 5% Na-TDC in the rat [123]. Among the various findings are impaired intestinal permeability, bacterial translocation, the perturbation of energy metabolism in the intestinal wall, pulmonary endothelial barrier dysfunction, microvascular endothelial barrier dysfunction, activation of mast cells, induction of nitric oxide synthase, increased catalytic activity of phospholipase A, increase cytokine levels, and increase myeloperoxidase activity.

Among bile salts, taurine-conjugated bile salt (Na-TC) is the most widely used and best characterized to date in inducing AP [123]. Experimental pancreatitis induced by Na-TC in the rat has represented the reference biliary AP for many years. Wu et al. (1991) reported that intraductal injection of Na-TC into the rat pancreas causes a disease with gross and histopathological changes that correspond to those seen in human pancreatitis [132]. The development of pancreatic changes was highly rapid. The experimental animals'mortality depended on the injected taurocholate concentration. The authors reported that the first histological change in acute bile-induced pancreatitis was the dissolution of the duct walls, with destroyed adjacent lobules. These lesions were at first patchy, but later, larger necrotic areas were seen. When the proteolytic enzymes are activated in the later phases, the lesions may spread to lobular regions not initially reached by taurocholate [132]. The necrotic areas never compromise the whole pancreas in this study. In 2002, Wu et al. reported that pancreatic subcapsular injection of 3.8% Na-TC could establish an AP rat model with multiple organ dysfunction but 90% mortality in a week. Liu et al. (2009) used a laparoscopic subcapsular injection of 3.8% Na-TC in rats with higher weights than Wu et al. and successfully established a model of AP [133]. The serum amylase activity increased significantly 6 h after induction, reaching a peak at 24 h and remaining at a higher level 48 h later. Pancreatitis pathology showed interstitial edema of the pancreas 6 h after model induction, with local hemorrhaging and slight inflammatory infiltration 48 h later [133]. The pancreatic pathology score on the 4^th^ and 7^th^ days showed an apparent phenomenon of necrotic pancreatitis, including interstitial edema, leukocyte infiltration, acinar cell necrosis, and hemorrhage [133, 134]. Gut barrier damage was observed as microvillus atrophy and intercellular junction disruption with bacterial translocation.

Laukkarinen et al. (2007) established a non-lethal biliary-AP model in mice using a retrograde ductal infusion of 50 ul 2% Na-TC. This protocol produced necrotizing pancreatitis in the head of the pancreas without associated lung injury [135]. Ductal infusions at 2 ml/kg of 4 and 5% Na-TC resulted in necrotizing pancreatitis, lung injury, and increased cytokines, causing 10% and 60% mortality rates at 24 h, respectively [136]. Wittel et al. (2008) describe successfully adapting the Na-TC model for severe pancreatitis from rats to mice. They developed a reliable and reproducible mouse model of severe necrotizing pancreatitis with a defined starting point for the onset of pancreatitis and pancreatitis-induced lethality [137]. They compared the Na-TC model to the most frequently used model of Cae-induced acute pancreatitis. They concluded that it resembles a model of locally induced pancreatic damage without systemic action of the pancreatitis-inducing agent.

9. **Post-Endoscopy Retrograde Cholangiopancreatography (ERCP)**- Endoscopic retrograde cholangiopancreatography (ERCP) is a specialized endoscopic procedure for managing pancreaticobiliary disorders. It has evolved to be a predominantly therapeutic tool, although ERCP carries a higher potential for complications than other endoscopic examinations. Post-ERCP pancreatitis (PEP) is one of the most frequent complications of ERCP, with an incidence of 1.5 to 15% [138]. However, it is not known why some patients develop AP, although several potential mechanisms of pancreatic injury during ERCP, including mechanical, thermal, chemical, hydrostatic, and enzymatic insults, among others, could be present. Kivisaari (1979) reported that contrast agents intraductally injected into the pancreatic duct provoked four days after AP with pancreatic atrophy, edema, leukocytosis, necrosis, and hemorrhage in rats [139]. A model of PEP based on high pressure (50 mmHg) of contrast agent intraductal injection produced histology alteration, edema, myeloperoxidase activity, and hemorrhage. In rat pancreas 24 h after injection [140]. The model mimicked the procedure of ERCP with meglumine. Contributing factors to the injury may include chemical toxicity from the contrast agent, disruption of pancreatic ducts, and even a rupture of acinar lobules due to forceful injection of contrast agent [45]. Other studies show that intraductal regulation of pH [141] and mechanical damage caused by direct papillary trauma [142, 143] affect the onset of AP and could be an essential factor in PEP. Ni et al. 2023 have developed a novel model of PEP in mice and reported that intraductal delivery of radiocontrast agent and calcineurin inhibitors was safe and well-tolerated with no significant acute or subacute endocrine or systemic toxicities, underscoring its clinical utility to prevent PEP [144].
10. **Ischemia/Reperfusion Acute Pancreatitis Model**- The role of ischemic injury as a cause of AP is recognized. Some studies have shown a correlation between the impairment of pancreatic microcirculation and the degree of ischemic injury [145–153]. Clinical and experimental studies have shown that ischemic injury plays an important part in the pathogenesis of acute pancreatitis [147, 148]. Ischemic damage to the pancreas is known to occur in various clinical settings. Alterations in the vascular bed of the pancreas or disturbances of the blood coagulation system are likely sequelae of acute pancreatitis. However, impairment of the pancreatic blood supply can, per se, lead to acute hemorrhagic pancreatitis. Redha et al. (1990) injected 20 microns of polystyrene microspheres retrogradely into the distal splenic artery of rats, thus incompletely blocking blood perfusion in the splenic portion of the pancreas. All the rats subjected to microsphere injection developed acute hemorrhagic pancreatitis by 27 h after surgery [153]. The authors concluded that acute experimental pancreatitis could be induced by partially blocking the arterial blood supply within the pancreas. Hoffmann et al. (1995) developed a model of complete ischemia/reperfusion of the pancreas in rats after interruption of arterial blood supply and reversible ischemia of the pancreas using intravital fluorescence microscopy [152]. The characteristics of the ischemia/reperfusion AP model were like those observed in the Na-TDC pancreatitis model. The severity of changes depends on the duration of ischemia and the duration of reperfusion. The morphological and biochemical modifications suggest that ischemia/reperfusion causes an inflammatory reaction, as observed in AP. The major studies, and complete and reversible ischemia, limited to ex vivo models [152]

## Conclusions

We analyzed the main experimental models of AP, summarized in Table 1; some of them are invasive and others non-invasive, which are carried out in different animals, but all of them are essential since they help us to evaluate the critical points in the development of the disease. Each model has advantages or disadvantages. To avoid the limitations and maximize the benefits of each model, improvements and combinations of the models have been made to reproduce human disease. Animal models have provided and continue to provide key insights into the etiology and pathogenesis of AP and aid in identifying new therapeutic targets that could be useful for treating the disease. However, only some of the existing AP models are currently acceptable. Still, new and more relevant models will be developed in the future, allowing us to get closer to having a response mechanism to what happens in humans.

**Table 1:** Note: This data is mandatory. Please provide

Model	Advantages	Disadvantages	Translational Medicine
Non-Invasive models			
Cae-induced or Hormone-Induced	The cerulein (Cae) model is among several animal models of experimental acute pancreatitis (AP) Suitable for investigating pathogenesis and cellular changes in early phases of AP The advantages are noninvasiveness, low cost, rapid induction, high reproducibility, and broad applicability	The induction of acute pancreatitis is performed by several injections Does not mimic severe form of human AP	Exhibit biochemical, morphological, and pathophysiological similarities to various aspects of human pancreatitis [31] Cae pancreatitis is like human edematous pancreatitis, manifesting with dysregulation of digestive enzyme production and cytoplasmatic vacuolization[31, 32]
Alcohol	The alcohol-induced model could be performed via intravenous, oral, intraperitoneal, and intraductal administration Alcohol increases pancreatic duct permeability, decreases pancreatic blood flow and microcirculation, diminishes pancreatic oxygen consumption, and induces oxidative stress	The morphology of the AP varies according to the animal model, the dose of ethanol administration, and the pre-sensitization agents used Acute pancreatitis induced by alcohol alone has been challenging to achieve and requires prior sensitization with other factors, such as exocrine hyperstimulation, to allow significant pancreatic damage to occur, so allows study of alcohol’s role in predisposing to AP	Alcohol is one of the major etiologic factors of acute pancreatitis [52] Allows studying the effect of toxic metabolites derived from oxidative and non-oxidative metabolism
Hypertriglyceridemia (HTG)	Models for establishing inducible HTG-AP must be induced through the diet	The TG levels will fluctuate with time after the model has been established	Hypertriglyceridemia-induced acute pancreatitis (HTG-AP) has become the second major cause of acute pancreatitis [73] HTG-AP that can mimic the physiological, histological, and clinical features of human HTG-AP [75]
Diet-Induced	Mimics severe human pancreatitis Choline-deficient diet diet has been associated with the activation of intrapancreatic proteases and characteristic vascular changes	Costly diet Requires standardization and careful monitoring of diet intake Depends critically on the sex, age, and weight of the mice Variation among animals could be high, making using many animals in each group necessary for meaningful results	This diet model is particularly suitable for studying the potential for new therapeutic substances as CDE diet-induced pancreatitis shares many characteristics with severe human acute pancreatitis [94]
Basic Amino Acid-Induced	Highly reproducible The degree of severity could be controlled as it depends upon the dose of arginine and time of exposure Interstitial edema, neutrophil infiltration, acinar cell degranulation, and elevated amylase are observed when arginine is administrated in low doses L-ornithine are simple, noninvasive, reproducible model of acute necrotizing pancreatitis by intraperitoneal injection L-lysine administration causes early pancreatic mitochondrial damage that impairs the ATP synthase activity of pancreatic mitochondria L-lysine model is economical and reproducible	Higher doses (7.5 g/kg) of L-arginine can be lethal for experimental animals, whereas a 2.5 g/kg dose only causes mild pancreatic injury L-arginine model its lack of clinical relevance AP caused by amino acid overdose is rare in humans	L-ornithine showing typical laboratory and morphologic signs observed in the human disease [96]
Invasive models			
Pancreatic Duct Ligation (PDL)	Mimicks gallstone-induced AP The surgical manipulation is simple, requiring either ligation of the common biliopancreatic duct Early changes in the duct can be observed at 1–5 h after ductal ligation, and 24 and 48 h findings such as pancreatic edema, inflammatory cell infiltration, and hyperamylasemia This cell model could identify acinar cell necrosis as the early point of injury in acute necrotizing pancreatitis	The complexity, technical difficulty, high cost, limited reproducibility, and the analogy to chronic pancreatitis have made the duct ligation model infrequently used for investigating AP	The duct obstruction model mimics gallstone obstruction-induced AP in the clinical setting. [22] This model mimics gallstone obstruction-induced AP with high mortality; thus, it could be used to investigate the pathogenesis of severe AP and test new therapies [45] Gallstone obstruction is the major etiologic factor of acute pancreatitis [121]
Bile Acid Salts-Induced	Short period needed to induce severe necrosis Impaired intestinal permeability, bacterial translocation, the perturbation of energy metabolism in the intestinal wall, pulmonary endothelial barrier dysfunction, microvascular endothelial barrier dysfunction, activation of mast cells, induction of nitric oxide synthase, increased catalytic activity of phospholipase A, increased cytokine levels and increase myeloperoxidase activity Is a model of locally induced pancreatic damage without systemic action of the pancreatitis-inducing agent	Requires abdominal incision and expertise in cannula insertion The experimental animals'mortality depended on the injected taurocholate concentration	Biliary calculous is the most common cause of AP and is associated with significant morbidity and mortality [121] Intraductal injection of Na-TC into the rat pancreas causes a disease with gross and histopathological changes that correspond to those seen in human pancreatitis [129]
Cholangiopancreatography (ERCP)	Is based on high pressure (50 mm Hg) of contrast agent intraductal injection produced histology alteration, edema, myeloperoxidase activity, and hemorrhage Endoscopic retrograde cholangiopancreatography (ERCP) is a specialized endoscopic procedure for managing pancreaticobiliary disorders	This model is very invasive and difficult to carry out Expensive	Post-ERCP pancreatitis (PEP) is one of the most frequent complications of ERCP, with an incidence of 1.5 to 15% [145]
Ischemia/Reperfusion model	It induces a partial blocking of the arterial blood supply within the pancreas Ischemia/reperfusion causes an inflammatory reaction, as observed in AP	The severity of changes depends on the duration of ischemia and the duration of reperfusion	Clinical and experimental studies have shown that ischemic injury plays an important part in the pathogenesis of acute pancreatitis [144.145]

## Data Availability

No datasets were generated or analysed during the current study.
