# Correction: Acute pancreatitis experimental models, advantages and disadvantages

**DOI:** 10.1007/s13105-025-01102-w

**Published:** 2025-06-18

**Authors:** Genaro J. Rosales-Muñoz, Verónica Souza-Arroyo, Leticia Bucio-Ortiz, Roxana U. Miranda-Labra, Luis E. Gomez-Quiroz, María Concepción Gutiérrez-Ruiz

**Affiliations:** 1 Departamento de Ciencias de La Salud, Área de Medicina Experimental y Traslacional, Universidad Autónoma Metropolitana-Iztapalapa, Mexico City, Mexico; 2 Laboratorio de Medicina Experimental, Unidad de Medicina Traslacional IIB/UNAM, Instituto Nacional de Cardiología Ignacio Chávez, Mexico City, Mexico

**Journal of Physiology and Biochemistry**

10.1007/s13105-025-01091-w.

This article has been published with incorrect figure captions.

The correct captions of all affected figures are as follows:

Figure 1.- Pathophysiology of acute pancreatitis.

The most frequent etiological agents that trigger the development of Acute Pancreatitis (AP) are biliary cholelithiasis, alcoholism, smoking, hypertriglyceridemia, and obstruction of the pancreatic duct. Regardless of the mechanism, the pathophysiology of AP leads to a deficiency in pancreatic excretion processes, preventing the exocytosis of zymogen granules (GZ) that contain digestive enzymes produced by acinar cells. Sustained cellular Ca2 + signaling leads to the development of aberrant exocytosis of GZ, which can co-localize with lysosomes in the plasma membrane, leading to intracellular activation of digestive enzymes that cause damage to different organelles such as the endoplasmic reticulum (ER), and mitochondria, causing reticulum stress (ER stress) and a persistent opening of the mitochondrial membrane permeability pore (MPTP), respectively. The secretion of chemoattractant factors causes the recruitment of immune system cells (neutrophils and macrophages) in the pancreas, which can promote tissue inflammation and culminate in a severe systemic inflammatory response. Created with Biorender.com.

Figure 2.- Acute pancreatitis animal models.

Animal models of acute pancreatitis (AP) are an essential research tool in translational medicine. The development of different experimental models allows the generating conditions analogous to the various etiologies that generate human AP. These can be divided into non-invasive models, which include those induced by hormones, alcohol, hypertriglyceridemia, choline-deficient diet, and basic amino acids ( L-arginine, L-Ornithine and L-lysine) as well as invasive models such as pancreatic duct ligation, intraductal bile salt injection, the endoscopic retrograde cholangiopancreatography (ERCP) model and the distal splenic artery ischemia/reperfusion model. Created with Biorender.com.

Figure 3.- Cellular pathogenesis of acute pancreatitis induced by cerulein.

The cerulein (Cae)-induced acute pancreatitis (AP) model is currently the most used model that resembles human AP. Cerulein (Cae) is a peptide analog of cholecystokinin (CCK); for instance, it can bind to cholecystokinin receptors (CCK-R) present in the membrane of pancreatic acinar cells; these receptors are classic seven transmembrane receptors coupled to trimeric G proteins (G proteins). Activation of these receptors leads to stimulation of Gαq and an increase in the activity of phospholipase C beta (PLCβ), which converts the membrane phospholipid: phosphatidylinositol,4,5-bisphosphate (PIP2) into diacylglycerol (DAG) and phospho-inositol. 1,4,5-triphosphate (PIP3). PIP3 interacts with PIP3 receptors (PIP3R), present in the endoplasmic reticulum (ER), releasing Ca2 + into the cytoplasm, which can induce the opening of the mitochondrial permeability transition pore (MPTP) and cause a decrease in ATP levels. In addition, an increase in the exocytosis of zymogen granules is also observed, causing their accumulation in the apical area of the acinar cell. This initiates the premature activation of digestive enzymes due to membrane colocalization between lysosomes and zymogen granules, ending in necrosis of the acinar cell. Created with Biorender.com.

Figure 4.- Cellular pathogenesis of acute pancreatitis induced by hypertriglyceridemia.

Hypertriglyceridemia (HTG) is considered the third cause of the development of acute pancreatitis. However, the mechanism by which HTG leads to the development of pancreatitis needs to be clarified; evidence suggests the following mechanism. The intestine absorbs dietary triglycerides (TG) and cholesterol (CHO), and they are transformed into chylomicrons (CM) that subsequently travel to the liver, where the hepatocytes package them into very low-density lipoproteins (VLDL), which will be captured by VLDL receptors (VLDLR) in the acinar cells. The lipoprotein lipases (LPL) in the acinar cell convert VLDL into TG and CHO; TG´s are the substrate of pancreatic lipase (PL) and lead to free fatty acids (FFA) as a product. When the regulation of FFA metabolism is inadequate, it can cause massive accumulation of FFA in the pancreas, which is cytotoxic per se, and trigger an inflammatory reaction, release intracellular Ca2+, and decrease ATP levels by opening the mitochondrial permeability transition pore (MPTP), and by the inhibition of mitochondrial complexes I and V, It can also affect the endoplasmic reticulum (ER), which in response to stress releases Ca2+, causing the aberrant release of zymogen granules, which can colocalize with lysosomes and the intracellular activation of digestive enzymes, which lead the acinar cell to necrosis. Created with Biorender.com.

